# The microbiome of an outpatient rehabilitation clinic and predictors of contamination: A pilot study

**DOI:** 10.1371/journal.pone.0281299

**Published:** 2023-05-04

**Authors:** Gabriella Brigando, Casey Sutton, Olivia Uebelhor, Nicholas Pitsoulakis, Matthew Pytynia, Thomas Dillon, Teresa Elliott-Burke, Nathaniel Hubert, Kristina Martinez-Guryn, Charlotte Bolch, Mae J. Ciancio, Christian C. Evans

**Affiliations:** 1 College of Dental Medicine–Illinois, Midwestern University, Downers Grove, IL, United States of America; 2 College of Health Sciences, Physical Therapy Program, Midwestern University, Downers Grove, IL, United States of America; 3 Physical Therapy Institute, Midwestern University Multispecialty Clinic, Downers Grove, IL, United States of America; 4 Independent Consultant, Bioinformatics Specialist, Chicago, IL, United States of America; 5 College of Graduate Studies, Biomedical Sciences Program, Midwestern University, Downers Grove, IL, United States of America; 6 Office of Research and Sponsored Programs, Midwestern University, Glendale, AZ, United States of America; University of North Dakota, UNITED STATES

## Abstract

**Background:**

Understanding sources of microbial contamination in outpatient rehabilitation (REHAB) clinics is important to patients and healthcare providers.

**Purpose:**

The purpose of this study was to characterize the microbiome of an outpatient REHAB clinic and examine relationships between clinic factors and contamination.

**Methods:**

Forty commonly contacted surfaces in an outpatient REHAB clinic were observed for frequency of contact and swiped using environmental sample collection kits. Surfaces were categorized based on frequency of contact and cleaning and surface type. Total bacterial and fungal load was assessed using primer sets specific for the 16S rRNA and ITS genes, respectively. Bacterial samples were sequenced using the Illumina system and analyzed using Illumina-utils, Minimum Entropy Decomposition, QIIME2 (for alpha and beta diversity), LEfSe and ANCOM-BC for taxonomic differential abundance and ADONIS to test for differences in beta diversity (p<0.05).

**Results:**

Porous surfaces had more bacterial DNA compared to non-porous surfaces (median non-porous = 0.0016ng/μL, 95%CI = 0.0077–0.00024ng/μL, N = 15; porous = 0.0084 ng/μL, 95%CI = 0.0046–0.019 ng/μL, N = 18. p = 0.0066,DNA. Samples clustered by type of surface with non-porous surfaces further differentiated by those contacted by hand versus foot. ADONIS two-way ANOVA showed that the interaction of porosity and contact frequency (but neither alone) had a significant effect on 16S communities (F = 1.7234, R^2^ = 0.0609, p = 0.032).

**Discussion:**

Porosity of surfaces and the way they are contacted may play an underestimated, but important role in microbial contamination. Additional research involving a broader range of clinics is required to confirm results. Results suggest that surface and contact-specific cleaning and hygiene measures may be needed for optimal sanitization in outpatient REHAB clinics.

## Introduction

Healthcare associated infections (HAIs) play a significant role in causing new illnesses and in prolonging the recovery process from previous illness and injury [1–3]. One indication of potential infections in a healthcare setting is the level of contamination by microbes on clinic surfaces or the collective “clinic microbiome”. While HAIs in general are carefully tracked, few studies have focused on contamination of physical therapy rehabilitation (REHAB) outpatient clinics [4–6]. Due to the extent to which patients and staff come in close contact with both clinic surfaces and each other, REHAB outpatient clinics may present an underappreciated source for transmission of infection. Identifying the outpatient REHAB clinic microbiome and determining how contamination occurs may help in the prevention of HAIs and in the development of protocols that better protect both healthcare providers and patients.

There has been recent concern about transmission of SARS CoV-2 on surfaces in healthcare settings, but a recent review that included 58 studies on indoor transmission of SARS CoV-2 suggested that while theoretically possible, there was little evidence of surface and fomite transmission [7]. Nevertheless, fomites and environmental surfaces are highly associated with bacterial and fungal pathogen transmission. Nosocomial bacterial infections are common in many hospitals and may arise from a variety of infectious sources [1, 3, 8]. One study found that 86.8% of sampled equipment within the hospital setting is contaminated with microbes [2]. The high rate of HAIs and high incidence of equipment contamination are in part attributed to hospitalized patients having multiple procedures, use of invasive devices, and the high incidence of patients taking antibiotics [2, 9]. However, it has been estimated that by adequately cleaning equipment, one-third of HAIs could be prevented and equipment contamination could be reduced by up to 80% [2]. Studies have found that the five most common HAIs cost $9.8 billion annually (data from 1986 to 2013) [10] with methicillin-resistant *Staphylococcus aureus* (*MRSA*) and *Clostridioides difficile* (*C*. *diff*) accounting for many of these costs [11].

Community-acquired and HAIs are closely linked. Studies have reported that the incidence of community acquired *MRSA* has been increasing [12]. Additionally, *MRSA* may develop in a setting removed from where it was initially acquired [9, 12]. According to Salgado et al., when patients known to be infected with nosocomial *MRSA* are discharged from the hospital, there is a high risk for spread to the community [9]. Moreover, it is likely some patients with MRSA go unrecognized while in the hospital and are discharged to outpatient services such as REHAB clinics, without knowledge of their potential to contaminate other facilities [9, 12]. This supports the need to carefully examine the level of contamination in healthcare facilities such as outpatient REHAB clinics, where patients coming from inpatient facilities are in contact with patients coming from the community.

A study conducted in an outpatient REHAB clinic indicated high level of contamination of clinic surfaces and equipment with microbes, including *MRSA*, and *Klebsiella pneumoniae* [4]. Spratt et al. examined outpatient rehabilitation clinic equipment, including ultrasound units and found gel bottles had the highest contamination level, with 52.7% positive for non-specific bacterial contamination and 3.6% positive for *MRSA* [5]. A study by Gontjes et al. examined contamination in the rehabilitation area of a nursing homes and tracked the transmission of microbes from surfaces to clients or staff [6]. This study found that 7.7% of equipment and 3.7% of hand samples were positive for multi-drug resistant bacterial strains. Additionally, they found that actual microbe transmission occurred in 17% of possible opportunities for transmission [6].

Given the potential for outpatient REHAB clinic environments to harbor microbes on surfaces and equipment and the potential to transmit these pathogens, it is important to determine what microbes are present and what factors promote contamination. The purpose of this study was to determine the extent of surface contamination, characterize the environmental microbiome and examine the relationship between the frequency of contact and cleaning and type of surface with the presence and types of microbes in an outpatient REHAB clinic. We hypothesized that surface contamination was primarily related to frequency of patient contact.

## Methods

**Fig 1:**
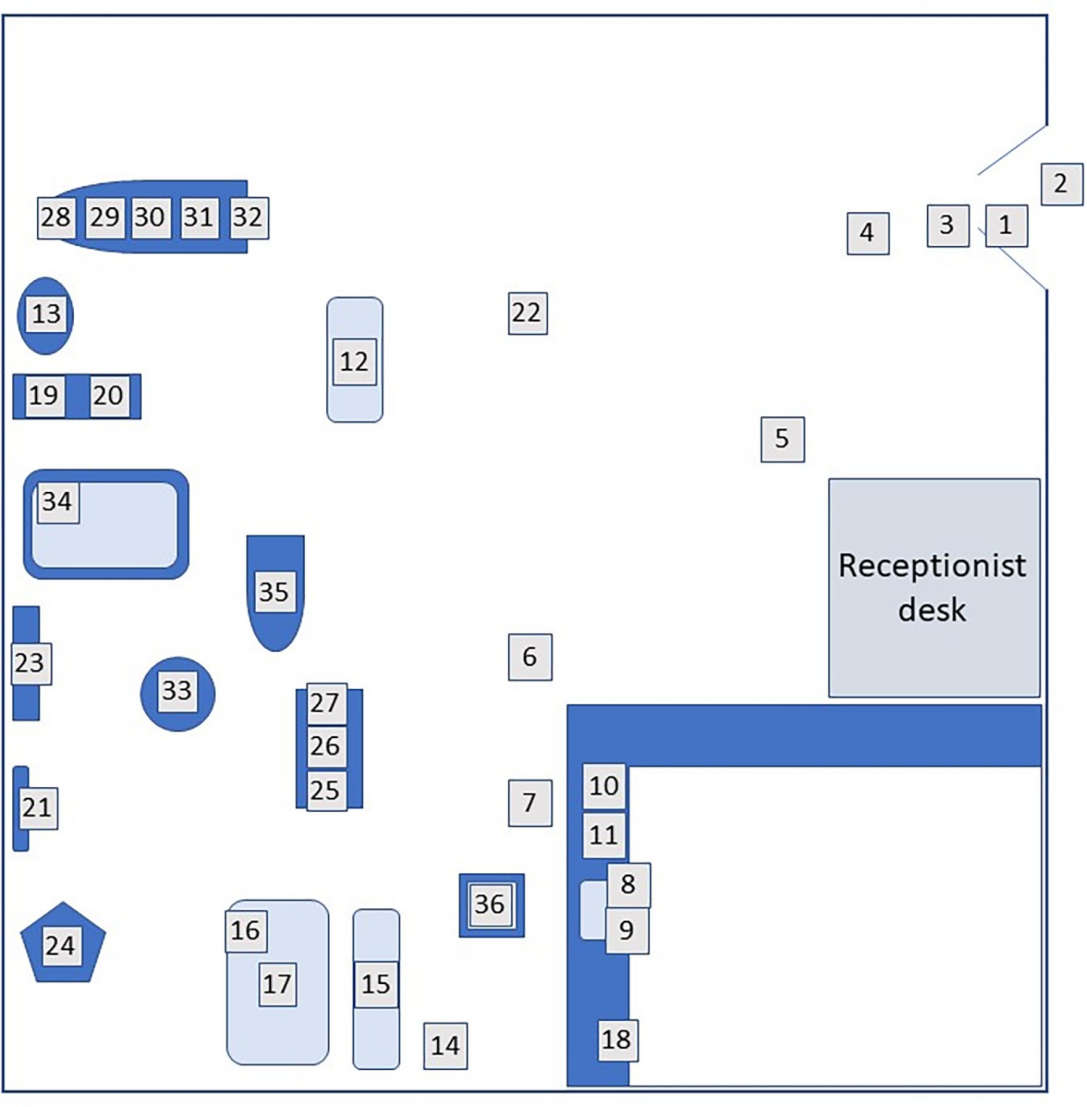
Map of the outpatient rehabilitation clinic. The sites that were observed for contact and swiped for collection of bacterial and fungal DNA are numbered and indicated. The clinic was approximately 14.5 M x 9 M in size and served approximately 10–15 patients/day during the data collection period. Typically, two physical therapists were present and treating patients and one administrative staff member was present. The clientele represented a wide range of diagnoses including low back and neck pain, knee, shoulder, ankle injuries, neurologic conditions and general medical conditions.

The REHAB clinic was a small (128.2 m^2^) outpatient facility in the Midwest, USA, that had a wide and varied referral base and provided services to a diverse patient population (combination of orthopedic, neurologic, and general medical diagnoses). The clinic was staffed by two full time clinicians. In order to survey the clinic microbiome, the most commonly contacted surfaces were first identified by a combination of discussion with the staff and by observation of the clinic. The REHAB clinic staff provided input as to which surfaces and equipment they contacted or used most frequently. A map of the clinic was used to develop a list of frequently contacted surfaces. This map was reviewed with the two primary clinic therapists at the site and their input was used to narrow down the list to the most frequently contacted surfaces (Fig 1). These 40 surfaces were then marked on the map with a brief description for three raters to reference during frequency of contact data collection. Frequency of contact was retrospectively binned into low (N = 5) and high (N = 31) total contacts. These surfaces were also carefully examined for the type of surface (porous versus non-porous material). The frequency with which each surface was cleaned and cleaning agent was determined by discussion with the clinic staff.

Type of surface material was dichotomized into non-porous (smooth, hard and non-penetrable) and porous (rough, cracked, foam, soft and penetrable surfaces). No published methods were available describing quantification of porosity or binning of surfaces by porosity for clinical surfaces; therefore, surfaces were classified by visual inspection. Non-porous surfaces included polished metal, vinyl floor tiles, counter surfaces, sink hot and cold handles and varnished wood. Porous surfaces included foam mats, untreated wood, treatment mats, rubber balls (with grooved surface), and unpolished rough surfaces such as the grips of weights and handles of the treadmill and bike. For frequency of cleaning, surfaces were binned into 3 categories: 1) low- those that were cleaned < once/day (floors, doorknobs, and bottom of the paper towel dispenser); 2) moderate- those that were cleaned at least once or twice/day, but not after every patient (counter tops, computer [keyboard and mouse] weights); and 3) high- those that were cleaned after every patient or 3 or more times/day (treatment mat and table, ultrasound/e-stim head, bike seat and handles). The type of cleaning agent was the same for all surfaces but was not considered in the analysis.

To determine the relationship between contamination of REHAB clinic surfaces with frequency of contact, the most commonly contacted surfaces were observed. Three raters (CS, OU and NP) observed the clinic to determine the frequency of surface contact by staff and patients and type of contact. In order to determine the inter-rater reliability, all three observed the clinic simultaneously on two separate days. Raters were instructed to not discuss their rating during data collection. Each rater tallied the number of times a surface was contacted (numbered by site) and reliability was determined by linear regression. Discrepancies were identified by reviewing all points and discussed by the raters to help minimize inconsistencies during future observations. Prior to collecting the swab samples for measurement of contamination, the clinic was observed to determine the frequency of contact of the identified sites. The three raters observed the clinic on different days of the week and at different times of the day to get a broad sample of the frequency of surface contact. The observations included a description of how the surface was contacted (patient or therapist and hand versus other body parts). During observations, patients and therapists were informed that a research project was in process but were not given details in order to avoid bias. Each session was generally 1–2 hours long and involved 1–2 patients, 1–2 therapists, and a receptionist. The frequency of contact was tallied for each rater and then combined to determine the total frequency of contact for each site based on who and how contacted. Interrater reliability was calculated by linear regression and reported as R^2^ value.

Surface swipe samples were gathered using specially designed synthetic porous swabs (Swab Collection and DNA Preservation System™) from Norgen Biotek Corp. over a one-hour break period in the middle of the day during a typical day at the clinic. Clinic staff were asked to go about their normal business and to perform their usual cleaning routine prior to collection (i.e., not to alter their cleaning procedure and not to do additional cleaning of surfaces). After staff and patients left the gym, a team of researchers swabbed the clinic using the environmental sample kits. Samples were placed into separate labeled sterile vials that were preloaded with a proprietary lysis buffer following the manufacturer’s instructions. Sample DNA was isolated and purified using a modification of the MasterPure™ Complete DNA and RNA Purification Kit (Epicentre, Inc.) as previously described [13]. Bacteria were lysed using zirconium beads and the Mini-Beadbeater^™^ (Biospec) for 3 minutes and then centrifuged. Impurities were removed by vigorously vortexing in a protein precipitating buffer. The supernatant was diluted with 100% isopropanol and allowed to stand overnight. DNA was pelleted by centrifugation x 10 min at 13,000 x g at 4°C. Isopropanol was removed without dislodging the pellet. The pellet was washed twice with 70% ethanol to further purify it and then resuspended in 40 μL of nuclease free water. DNA quality and purity were checked using the NanoDrop One/OneC Microvolume Spectrophotometers^™^ (Thermo Scientific, Inc). The range of concentrations of DNA was from 0.9–36.7 ng/μL.

Quantification of total bacterial and fungal DNA was based on total surface area swiped and not standardized to area (i.e., surface area differed between samples). The Femto DNA Quantification^™^ kits for total bacterial and total fungal DNA (Zymo Research, Inc) were used to quantify the amount of DNA from each kingdom of microbes, respectively, based on specific universally conserved regions of DNA. The bacterial kit used a universal 16S rRNA gene primer set, the fungal kit used a universal fungal ITS region and both used SYTO-9 fluorescent probes. These methods have been previously published [14, 15].

The same DNA samples used for qPCR quantification of total bacteria were sequenced on the Illumina MiSeq platform (Argonne National Laboratory, Institute for Genomics and Systems Biology, Next Generation Sequencing Core). Illumina-utils was used to align, merge and quality filter the sequence data. Minimum entropy decomposition (MED) [16] was used, allowing no mismatches between forward and reverse compliment 16S rRNA sequences, to rigorously define homogenous taxonomic nodes. Global Alignment for Sequence Taxonomy (GAST) [17] was used to assign taxonomy using the SILVA database (version 138) [18]. Alpha and Beta diversity were assessed at all levels and Beta diversity was assessed using principal coordinate analysis (PCoA) based on Bray-Curtis distances in QIIME version qiime2-2019.7 [19] and resulting ordinations were visualized using Emperor software [20]. Initial analysis was based on frequency of contact, type of surface and frequency of cleaning, but analysis by type of surface findings indicated a secondary factor, “how surfaces were contacted” (by hand or foot). Surface type and how contacted were explored through secondary analysis.

Linear discriminant analysis Effect Size (LEfSe) was used to test for differential abundances, and to visualize how phylogeny relates to taxa differentiated by treatment [21]. Bar plots were generated in LEfSe showing significantly differentiated taxa, i.e., taxa having an LDA score Log10 >2 or <-2 SD. Differential taxonomic abundances were also determined using Analysis of Compositions of Microbiomes with Bias Correction (ANCOM-BC) with R (RStudio Team 2020, Integrated Development for R. RStudio, PBC, Boston, MA) in QIIME2. The ANCOM-BC estimates the sampling fractions that are unknown and corrects for the bias introduced by the differences among samples. This analysis provides valid statistical tests while controlling the False Discovery Rate and maintaining adequate power [22]. Taxa that were determined to be significantly differentiated by both LEfSe and ANCOM-BC were considered robust markers of treatment differentiation. ADONIS (i.e. PERMANOVA) was used to compare communities by Bray-Curtis distances. Total bacterial and fungal DNA data was checked for normalcy with Shapiro-Wilk test. Analysis for outliers was performed using the ROUT method with Q = 1% (SPSS v27). Clustering of surface samples was examined by identifying grouping structures within the data based on frequency of contact. A K-means analysis was performed where the number of desired clusters was defined with n = 2 and n = 3 clusters. Maximum iterations were set to 10 and the convergence criterion was defined as 0. Convergence was achieved after 3 iterations with n = 2 due to no or small change in cluster centers. The final cluster centers and number of cases in each cluster were determined as the following: Cluster 1 (center = 7, n = 31) and Cluster 2 (center = 101, n = 5) (SPSS v27). Porous and non-porous, contact, and cleaning frequency groups were compared with Kruskal-Wallis on Prism 8 software (GraphPad Software, San Diego, CA) or using Kruskal-Wallis test in QIIME version qiime2-2019.7 [19]. Correlations between frequency of contact and level of contamination was determined by Spearman’s correlation. All comparisons were displayed as either mean ± SD or 95% confidence interval or as median ± interquartile range and maximum and minimum range. The criteria for significance was P < 0.05 in all comparisons and correlations.

## Results

A total of 40 surfaces were initially identified in the clinic. All 40 sites were swiped, but only 33 were included in quantification of total bacterial and 36 for fungal DNA due to insufficient DNA. The final 36 surfaces included in the analysis are shown in map form in Fig 1. The types of surfaces are identified in Table 1 along with a description of frequency of contact and cleaning, and surface type classification. Inter-rater reliability was determined by performing linear regression on frequency counts from three observers while simultaneously observing the clinic. The R^2^ for the three observers was 0.974, suggesting a high degree of correlation between observers and good inter-rater reliability.

**Table 1:** Clinic surfaces.

Site #	Description	Contact frequency	Contact category	Porosity category	Cleaning frequency	Total bacteria (ng/μL)	Total fungi (ng/μL)	
1	Outer door handle	2	Low	NP	Low	0.00306	0.000672	
2	0.3 m^2^ square in front of door	73	High	NP	Low	0.00028	0.000002	
3	Inner door handle	0	Low	NP	Low	0.00238	0.000523	
4	0.3 m^2^ square after entering	78	High	NP	Low	N/A	0.000003	
5	0.3 m^2^ square by desk	8	Low	NP	Low	0.00136	0.000293	
6	0.3 m^2^ square at corner countertop	62	High	NP	Low	0.00168	0.000841	
7	0.3 m^2^ square by sink	36	Low	NP	Low	0.02671	0.002753	
8	Entire sink hot handle	11	Low	NP	Low	0.00718	0.001071	
9	Enter sink cold handle	0	Low	NP	Low	0.00158	0.003134	
10	Button on soap dispenser	9	Low	P	Low	0.00351	0.001265	
11	Bottom of metal paper towel dispenser	10	Low	NP	Low	0.00235	0.001962	
12	Entire surface of yoga mat	0	Low	P	Low	0.00497	0.007736	
13	BAPS board	0	Low	NP	Low	0.00081	0.002211	
14	Foam roller	4	Low	P	Low	0.00195	0.00417	
15	Small high low Table 4 corners	137	High	P	High	0.00327	0.001482	
16	Big high low Table 4 corners	8	Low	P	High	0.00893	0.004607	
17	Small high low table center hole	12	Low	P	High	0.01737	0.004528	
18	Mouth and earpiece of phone	0	Low	P	Low	0.02413	0.000369
19	Adjustable weight bench lower section	3	Low	P	Mod	0.00716	0.00061	
20	Adjustable weight bench head piece	1	Low	P	High	0.00268	0.00018	
21	Handles of pulley system	33	Low	P	Mod	0.00013	0.000106	
22	Top of wooden frame step-up	4	Low	NP	Low	0.00001	0.000176	
23	Sample of small weight set	0	Low	P	Low	0.00586	0.001553	
24	Head of e-stim	2	Low	NP	High	0.00163	0.000066	
25	Upright bike seat	6	Low	P	Med	0.01547	0.001292	
26	Upright bike handle grips	12	Low	P	Med	0.0136	0.003444	
27	Upright bike foot plates	4	Low	NP	Med	0.01405	0.004906	
28	Total Gym™ sample of weights	0	Low	NP	Low	N/A	0.000525	
29	Total Gym™ top section	0	Low	P	Low	N/A	0.000059	
30	Total Gym™ bottom section	0	Low	P	Low	0.0004	0.000722	
31	Total Gym™ handles	0	Low	NP	Low	0.00075	0.000017	
32	Total Gym™ foot plate	0	Low	NP	Low	0.01807	0.000143	
33	Large rubber gym ball	17	Low	P	Low	0.01193	0.006587	
34	Treadmill- hand rails and screen	2	Low	P	Mod	0.01416	0.002242	
35	Recumbent bike seat and handles	32	Low	P	Mod	0.05658	0.003264	
36	Lap top computer-mouse	132	High	P	Mod	0.00956	0.000849	

Categories for frequency of contact were: Low and High based on clustering analysis with cluster centers at 7 (n = 31) and 101 (n = 5). Categories for type of surface were porous and non-porous. Categories for frequency of cleaning were low = < once/day, Moderate = one-two x/day but not after every patient, high = after every patient or ≥ 3 times/day. Bacterial and fungal DNA was expressed as ng DNA/μL. Abbreviations or equipment manufacturer names are as follows: BAPS, Biomechanical Ankle Platform System, Spectrum Therapy manufacturer; e-stim, electrical stimulator; m, meters; Mod, moderate; N/A, not available; ng, nanograms; NP, non-porous; P, porous; Total Gym™ Total Gym system-Global Corp; μL, micro liters.

Table 1 shows the frequency of contact, type of surface and frequency of cleaning along with total bacteria and fungi for all surfaces analyzed. From observations at the clinic, the two most frequently contacted surfaces over the 20-hour total observation period were #15, the small high-low mat table (137 times) and #36, the laptop (132 times). There were 9 surfaces that were not contacted at all which included #12, yoga mat, #18, phone and #13, the BAPS board.

Quantification of total bacterial and fungal DNA revealed approximately 75% more bacterial DNA than fungal DNA on surfaces (mean bacterial DNA = 0.0081 ±0.0019ng/μL vs. mean fungal DNA = 0.0018 ± 0.0003ng/μL, P = 0.0013). There was a low correlation (Pearson’s r = 0.302, P = 0.0778) between bacterial and fungal contamination on surfaces with all the data included, but when outliers (> 2 SD beyond mean for group) were removed, there was a fair correlation (Pearson’s r = 0.5147, P = 0.0036). Surfaces with the highest level of bacterial contamination included #35, the recumbent bike seat and handlebars (0.05658ng/μL total bacterial DNA) #7, the floor in front of the sink (0.02671ng/μL total bacterial DNA), and #18, the mouth and earpiece of the phone (0.02413ng/μL total bacterial DNA) (see Table 1 and Fig 1). Sites with very low levels of bacterial contamination included #21, handles of pulley system (0.00013ng/μL total bacterial DNA), #22, top of the wooden frame step (0.00001ng/μL total bacterial DNA) and #2, the 0.3 m^2^ square in front of door (0.00028ng/μL total bacterial DNA) (see Table 1 and Fig 1). Surfaces with the highest level of fungal contamination included #12, the yoga mat (0.00774 ng/μL total fungal DNA), #33, gym ball (0.006587ng/μL total fungal DNA) and #27, upright bike foot plates (0.004906ng/μL total fungal DNA). The lowest level of fungal DNA was detected at #2 and #4, floor directly inside and outside the entrance to the gym (0.000002 and 0.000003ng/μL, respectively).

**Fig 2:**
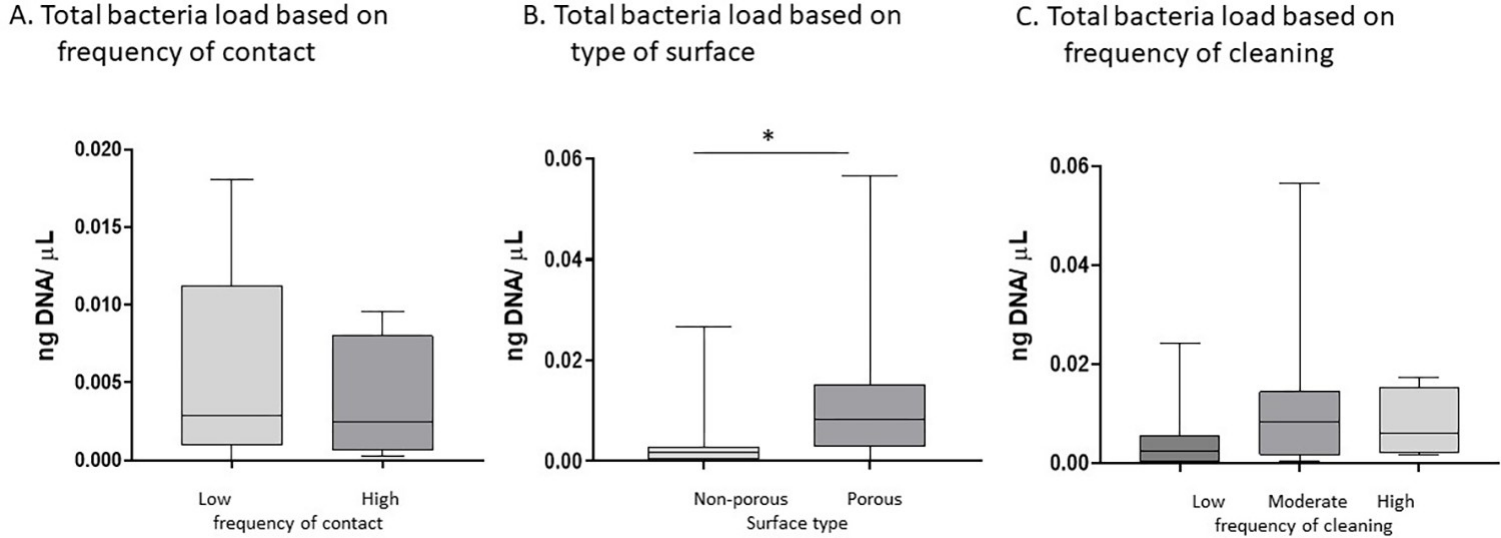
Bacterial contamination and clinic factors. Total bacterial DNA based on 16S rRNA gene quantification was analyzed. A. Total bacterial DNA comparing surface frequency of contact as low or high. B. Total bacterial DNA comparing surfaces identified as non-porous versus porous. C. Total bacterial DNA comparing surfaces with low, moderate and high frequency of cleaning. * indicates statistically significant difference. Abbreviations used are as follows: ng, nanograms; μL, microliters.

**Fig 3:**
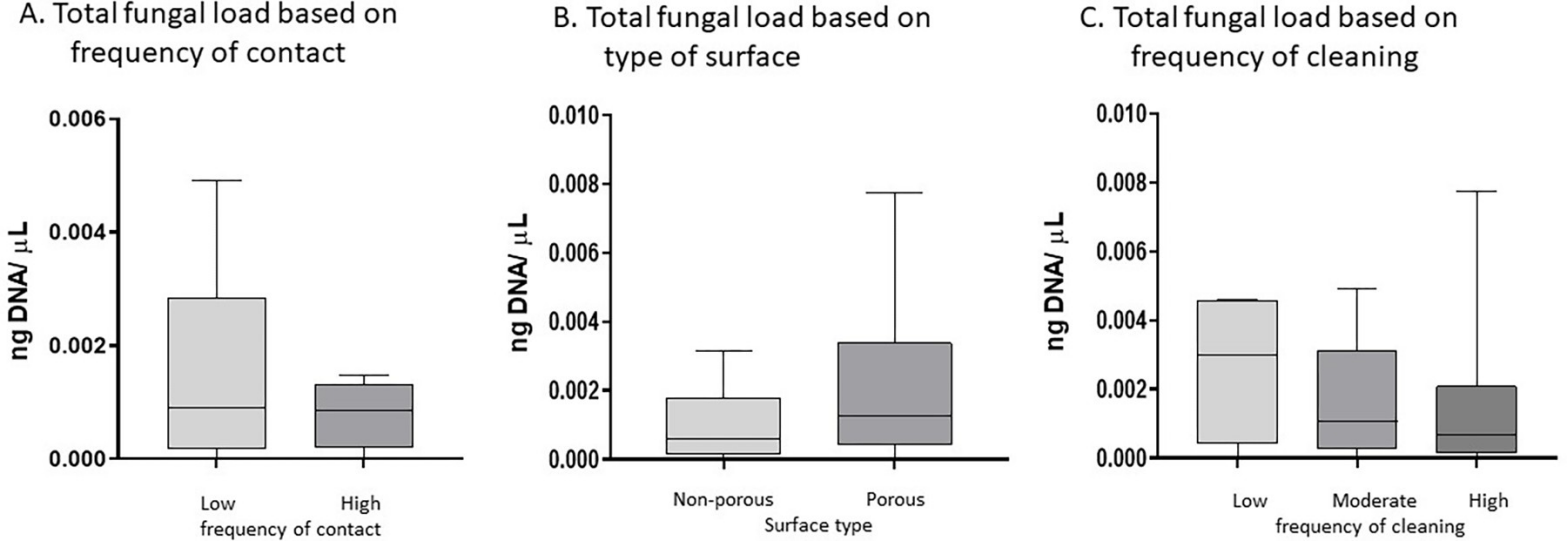
Fungal contamination and clinic factors. Total fungal DNA based on ITS gene region quantification. A. Total fungal DNA comparing surfaces with low or high frequency of contact. B. Total fungal DNA comparing surfaces identified as non-porous versus porous. C. Total fungal DNA comparing surfaces with low, moderate and high frequency of cleaning. * indicates statistically significant difference. Abbreviations used are as follows: Abbreviations used are as follows: ng, nanograms; μL, microliters.

Correlations between “frequency of contact” and total bacterial or fungal DNA showed that frequency of contact was not correlated with either bacterial (Spearman R = -0.05272, 95% CI = -0.3793–0.2855, p = 0.7636) or fungal contamination (Spearman R = -0.243, 95% CI = -0.5365–0.1030, p = 0.1533) (Table 2). Comparison of the total bacterial DNA based on frequency of contact, type of surface (porous versus non-porous) and cleaning frequency (low, moderate or high) showed that only classification by surface type significantly differentiated total bacterial DNA load. Porous surfaces had more bacterial DNA compared to non-porous surfaces (median non-porous = 0.0016ng/μL, 95%CI = 0.0077–0.00024ng/μL, N = 15; porous = 0.0084 ng/μL, 95%CI = 0.0046–0.019 ng/μL, N = 18. p = 0.0066, Fig 2). Comparison of the total fungal DNA based on frequency of contact or cleaning and type of surface showed that no grouping differentiated total fungal DNA load (Fig 3).

**Table 2:** Spearman correlations between bacterial and fungal contamination with frequency of contact.

Total bacteria	R value	95% Confidence interval	P value	Significance
Contact frequency	-0.05272	-0.3793 to 0.2855	0.7636	N/S
**Total fungi**				
Contact frequency	-0.243	-0.5365 to 0.1030	0.1533	N/S

Spearman correlation based on clinic factors using GraphPad Prism 5. P < 0.05 was criteria for significance in correlations. Abbreviations are as follows: N/S, non-significant.

**Fig 4:**
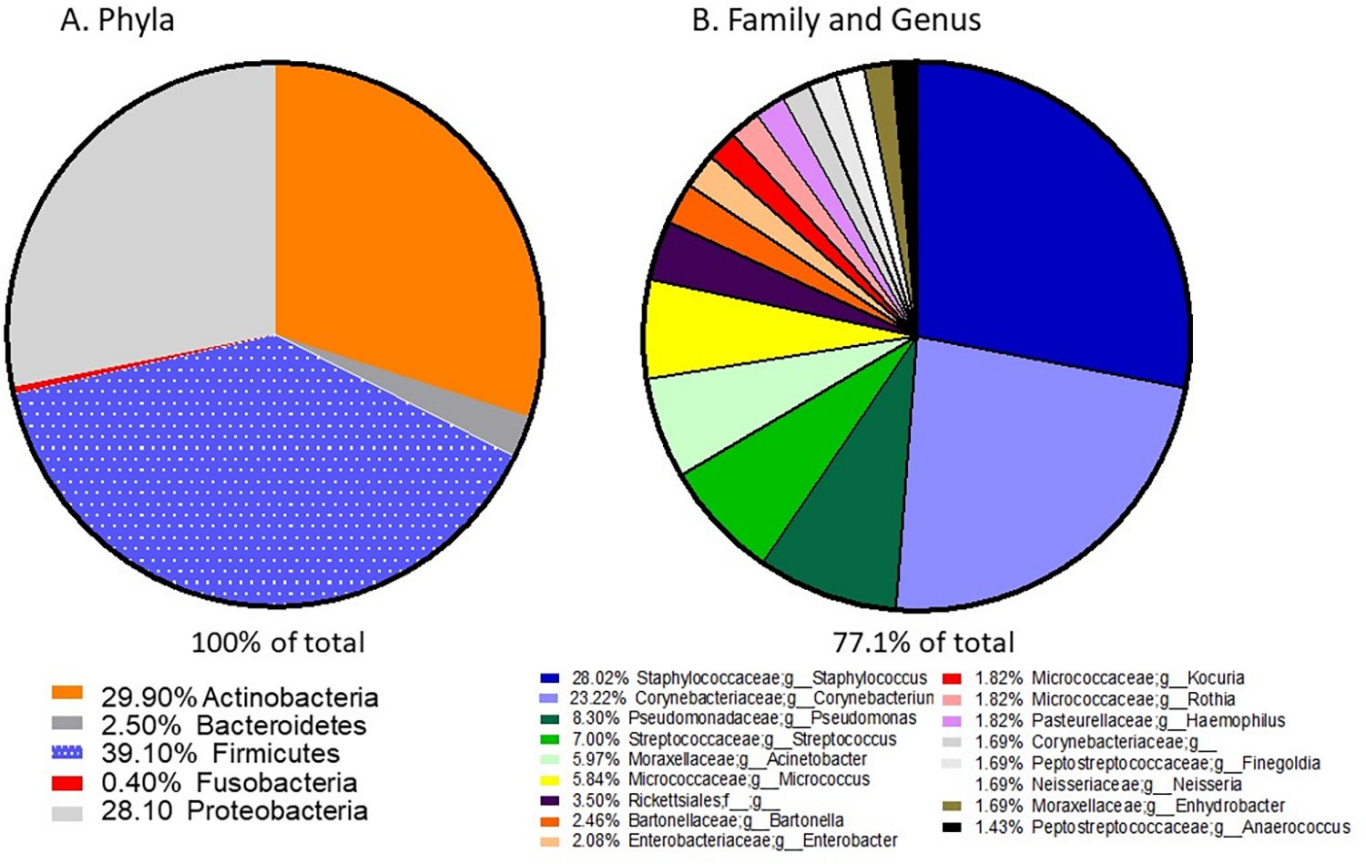
Clinic surface bacterial taxa. A. Major phyla. B. Family and genus. Bacterial taxa were determined by 16s RNA gene sequencing.

Analysis of the clinic microbiome by 16S rRNA gene sequencing was carried out and data is available from NIH Sequence Read Archive with Bioproject Accession #PRJNA889035 (https://www.ncbi.nlm.nih.gov/bioproject/PRJNA889035/). Results showed that the phyla *Firmicutes* composed 39.1%, *Actinobacteria* composed 29.9%, and *Proteobacteria* composed 28.1% of all bacteria present, totaling 97.1% of bacteria (Fig 4A). A smaller percentage of bacteria were *Bacteroidetes* (2.5%) and *Fusobacteria* (0.4%). At the genus level, *Staphylococcus* and *Corynebacterium* were most prevalent (28.02% and 23.22%, respectively), while *Pseudomonas*, *Streptococcus*, *Actinetobacter*, and *Micrococcus* composed 8.3%, 7%, 5.97% and 5.84%, respectively (Fig 4B). Although present at low levels, *Bartonella*, *Enterobacter*, *Haemophilus*, and *Neisseria* were detected (2.46%, 2.08%, 1.82% and 1.69%, respectively).

**Fig 5:**
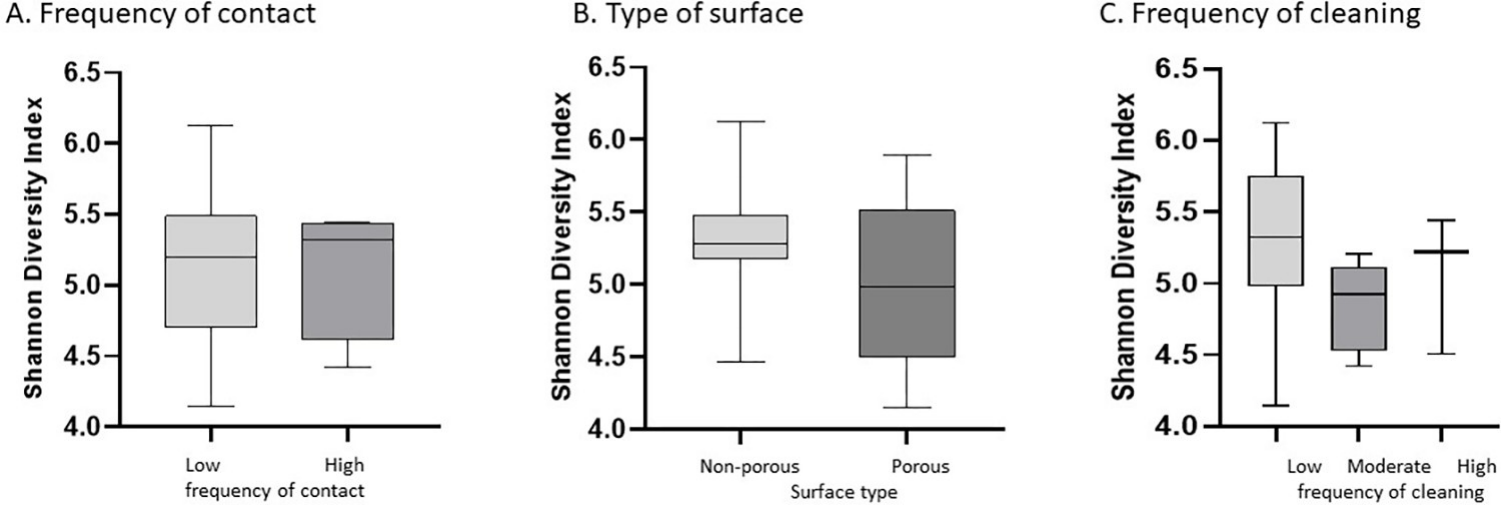
Alpha diversity based on Shannon Diversity Index of clinic surface microbiome. A. Shannon Diversity for surfaces with low or high frequency of contact. B. Shannon Diversity for non-porous and porous surfaces. C. Shannon Diversity for surfaces based on low, moderate and high frequency of cleaning. No differences were detected based on any of these groupings. Abbreviations used are as follows: Mod = moderate.

Alpha and Beta diversity are presented at the genus and oligotype level, respectively, but limited to a subset of samples. The cutoff for minimal reads was 5,000; therefore, nine samples (# 6, 9, 17, 24, 27–29, 31 and 33) were not included for further analysis. Results showed that grouping by frequency of contact or cleaning as well as by surface type did not differentiate based on alpha diversity (Fig 5A–5C). However, when these factors were taken together, there was a significant effect of the combination of contact frequency and surface type on 16S beta diversity as assessed by ADONIS two-way analysis (Table 3).

**Table 3:** Two-way ADONIS correlation analysis of clinic factors based on oligotype variability.

Clinic factor	DOF	SOS	Mean SOQ	F statistic	R^2^	P value
Surface porosity	1	0.2326	0.2326	1.4217	0.0503	0.092
Contact frequency	2	0.3492	0.1746	1.0675	0.0755	0.35
Surface porosity * contact frequency	4	0.2819	0.2819	1.7234	0.0609	0.032*
Residuals	23	3.7623	0.1636	N?A	0.8133	N/A

ADONIS analysis based on clinic factors using QIIME distance data at the oligotype-level. Frequency of contact was categorized as low and high frequency and porosity was dichotomized as porous or non-porous. Frequency of cleaning did not contribute to the model and was not included in the final analysis. P < 0.05 was criteria for significance in correlations. Abbreviations used were as follows: *, statistically significant interaction; DOF, degrees of freedom; SOS, sum of squares.

**Fig 6:**
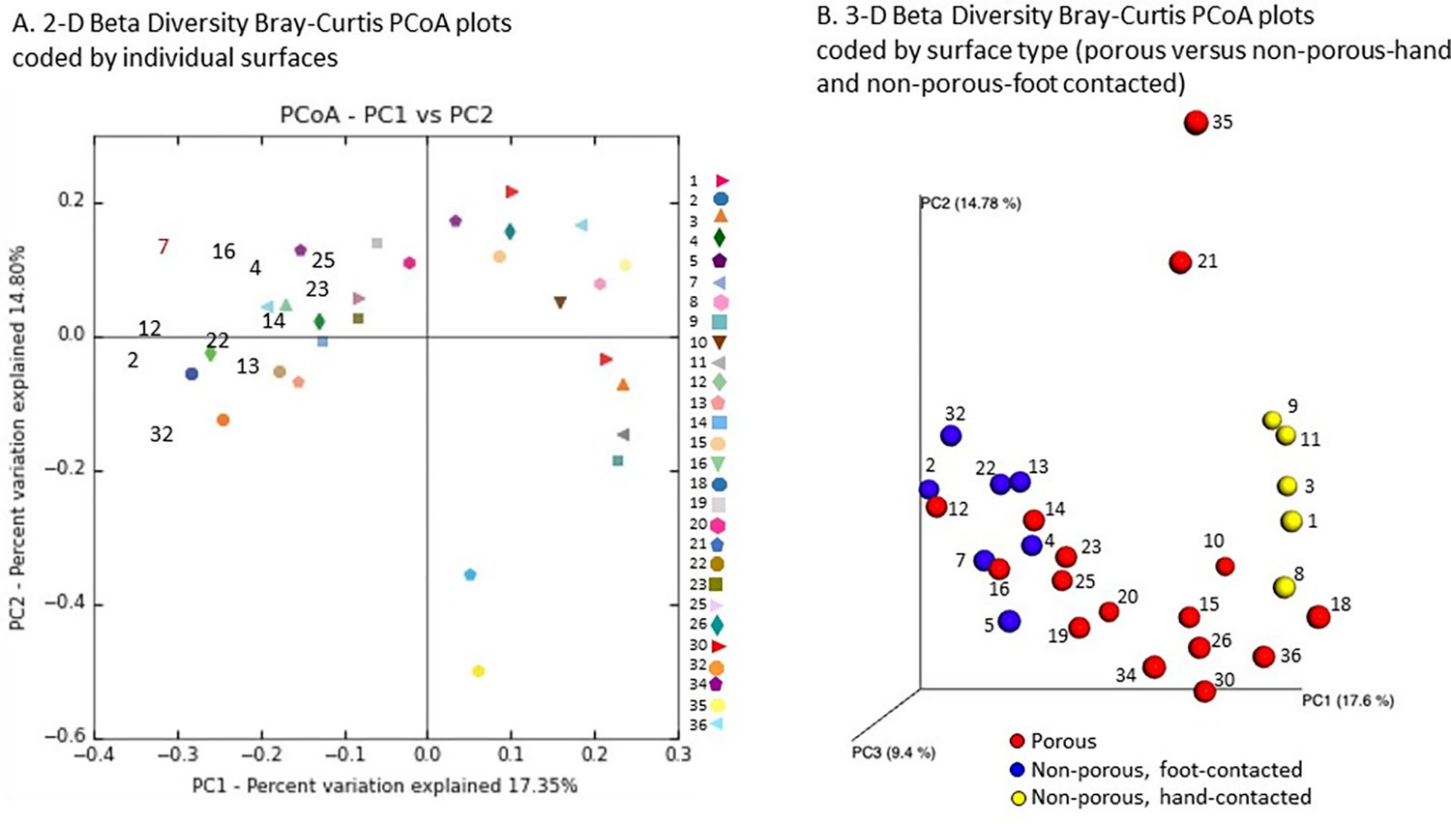
Beta diversity of clinic surface microbiome. A. Beta diversity on a two-dimensional principle coordinate analysis plot with all surfaces samples marked individually. No clustering can be detected based on subjective analysis of the plot. B. Beta diversity on a three-dimensional plot with surfaces coded based on classification of surfaces as non-porous-hand contacted, non-porous-foot contacted and porous. In this plot there is separation of the samples coded red (porous), blue (non-porous, foot contacted) and yellow (non-porous, hand-contacted) along the X-axis. Only 28 of the 36 samples were include and samples 6, 17, 24, 27, 28, 29, 31, and 33 could not be analyzed due to insufficient reads. Abbreviations are as follows: PC = principal coordinate; PCoA = principal coordinate analysis.

The overall beta diversity was assessed using Bray-Curtis distances. Fig 6A shows the Bray-Curtis two dimensional PCoA ordination for the 28 data points that met the minimal read threshold for analysis. Coding data points based on frequency of contact (both for total contacts as well as patient or therapist contact) and frequency of cleaning revealed no specific clustering patterns; however, when examined based on type of surface, there was clustering and a bimodal distribution of the non-porous surfaces (combination of blue and yellow) to either side of the porous surfaces (center) on the primary axis (PC1) axis in 3-dimensional Bray-Curtis PCoA display (Fig 6B). This bimodal distribution suggested that the non-porous sub-samples were different with respect to the microbial environment. Identification of the samples in Fig 6B revealed that the non-porous (blue) on the left were primarily associated with contact by foot (#2, floor in front of main entrance, #4, floor just inside door, #5, floor in front of desk, #7, floor by sink, #13, foot plate of BAPST board, #22, top of wooden step, and #32, foot plate of Total Gym). Alternately, the non-porous samples on the right (yellow) were primarily associated with hand contact (#1, outer door handle, #3, inner door handle, #8, sink hot handle, #9, sink cold handle, #11, bottom of paper towel dispenser). Identification of the porous samples did not reveal any additional clustering patterns.

**Fig 7:**
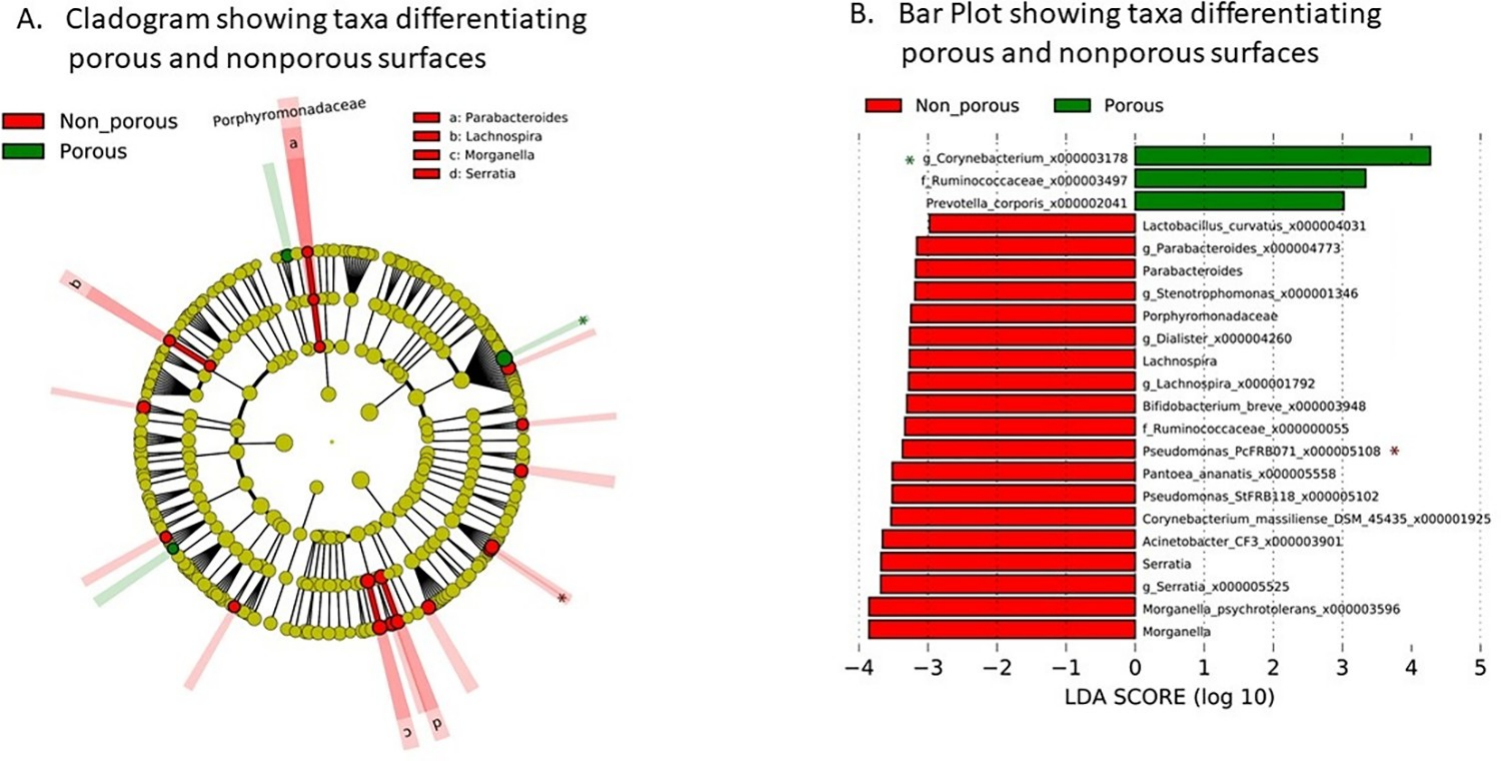
A. Cladogram representing the taxonomy of 16S rRNA amplicons found in this study differentiated by porous versus non-porous surface type. Green indicates taxa significantly more abundant in porous samples, whereas red indicates taxa more abundant in non-porous samples. Prominent taxa are identified as nodes and indicated in the figure or key. B. Bar chart showing the oligotypes identified by LEfSe analysis that were enriched in porous (green) and non-porous samples (red). Asterisks indicate taxa that were found to be significantly differentiated by LEfSe. Abbreviations are as follows: LDA, linear discriminate analysis score.

**Fig 8:**
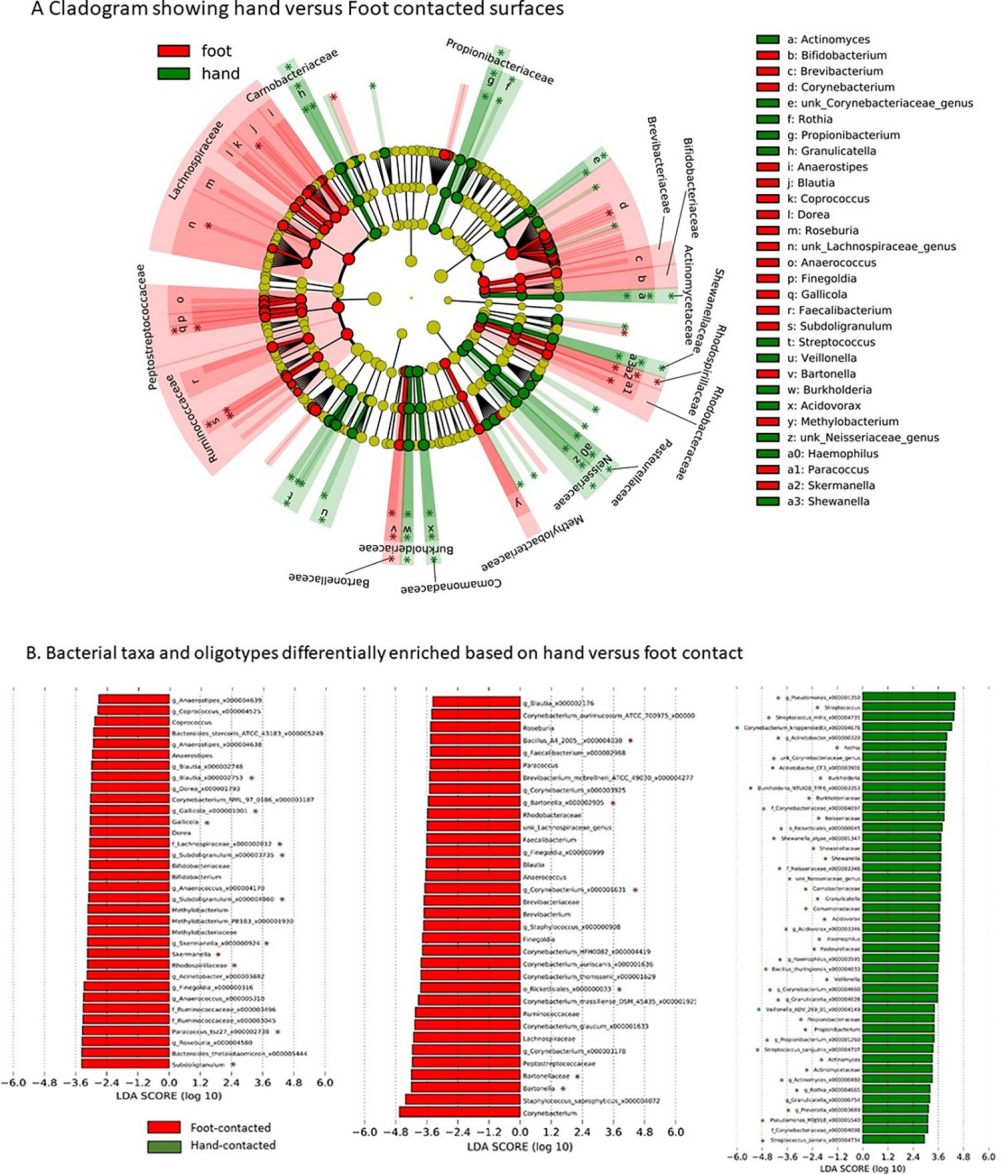
A. Cladogram representing the taxonomy of 16S rRNA amplicons found in this study differentiated by hand versus foot-contacted surfaces. Green indicates relationships for hand-contacted and red indicates foot-contacted surfaces. B. Bar chart showing the oligotypes identified by LEfSe analysis that were enriched in hand (red) and foot-contacted surfaces (green). Abbreviations are as follows: LDA, linear discriminate analysis score. Asterisks indicate taxa that were found to be significantly differentiated by both LEfSe and ANCOM-BC.

The LEfSe and ANCOM-BC analyses revealed differences in the abundance of multiple 16S taxonomic nodes based on surface type and how contacted (Figs 7A and 8A). For porous and non-porous samples, ANCOM-BC found that a *Corynebacterium* oligotype (i.e., homogenous taxonomic unit derived from MED analysis of 16S rRNA sequences) was enriched in porous (W = 3.68951, p = 0.00022, q = 0.04538), whereas a *Pseudomonas* oligotype was enriched in non-porous samples (W = -4.0073, p = 0.0006, q = 0.01237) (Fig 7B, Table 4). Based on ANCOM-BC, 11 different oligotypes were enriched in foot-contacted surfaces as shown in Table 4. The 5 oligotypes most enriched in foot-contacted surfaces were identified to the most resolved taxonomy possible using GAST, belonging to *Rickettsiales* (W = -9.93595, p < 0.001, q < 0.001), *Paracoccus* tsz27 (W = -9.44017, p < 0.001, q < 0.001), *Bacillus A4*(2005) (W = -6.01017, p < 0.001, q < 0.001), *Lachnospiraceae* (W = -4.85865 p < 0.001, q < 0.001), and *Bartonella* (W = -4.83700 p < 0.001, q < 0.001). Based on ANCOM-BC, 23 different oligotypes were enriched in hand-contacted surfaces. The 5 oligotypes most enriched in foot-contacted surfaces were most resolvedly identified to *Streptococcus* mitis (W = 11.30945, p < 0.001, q < 0.001), *Corynebacterium* kroppenstedtii (W = 8.33584, p < 0.001, q < 0.001), *Corynebacteriaceae* (W = 7.84495, p < 0.001, q < 0.001), *Streptococcus* sanguinis (W = 7.49122, p < 0.001, q < 0.001) and *Actinomyces* (W = 7.09812, p < 0.001, q < 0.001). All of the oligotypes found to be enriched by ANCOM-BC were also identified as enriched using the LEfSe technique (Fig 8B).

**Table 4:** Bacterial oligotypes differentiating surfaces.

	Porous versus non-porous surfaces					
Type of surface				W statistic	p value	q value
Non-porous	*Pseudomonas* PcFRB071_x000005108		-4.00730	0.00006	0.01247
Porous	genus *Corynebacterium*_x000003178		3.68951	0.00022	0.04538
	**Hand versus foot-contacted surfaces**				
		Type of contact		W statistic	p value	q value
	Foot		order Rickettsiales_x000000033	-9.93595	0.00000	0.00000
		Foot	*Paracoccus* tsz27_x000002738	-9.44017	0.00000	0.00000
		Foot	*Bacillus* A4(2005)_x000004030	-6.01017	0.00000	0.00000
		Foot	family *Lachnospiraceae*_x000002012	-4.85865	0.00000	0.00024
		Foot	genus *Bartonella*_x000002905	-4.83700	0.00000	0.00027
		Foot	genus *Skermanella*_x000000924	-4.48311	0.00001	0.00146
	Foot		genus Gallicola_x000001001	-3.98539	0.00007	0.01300
		Foot	genus *Subdoligranulum*_x000003735	-3.93234	0.00008	0.01615
		Foot	genus *Subdoligranulum*_x000004860	-3.86125	0.00011	0.02155
		Foot	genus *Blautia*_x000002753	-3.66470	0.00025	0.04606
		Foot	genus *Corynebacterium*_x000001631	-3.66105	0.00025	0.04647
		Hand	*Streptococcus mitis*_x000004735	11.30945	0.00000	0.00000
		Hand	*Corynebacterium kroppenstedtii*_x000004678	8.33584	0.00000	0.00000
		Hand	family *Corynebacteriaceae*_x000004097	7.84495	0.00000	0.00000
		Hand	*Streptococcus sanguinis*_x000004707	7.49122	0.00000	0.00000
		Hand	genus *Actinomyces*_x000000480	7.09812	0.00000	0.00000
		Hand	genus *Acinetobacter*_x000000320	6.74829	0.00000	0.00000
		Hand	genus *Haemophilus*_x000003595	6.37992	0.00000	0.00000
		Hand	*Shewanella algae*_x000001347	6.02915	0.00000	0.00000
		Hand	genus *Rothia*_x000004665	5.92118	0.00000	0.00000
		Hand	genus *Propionibacterium*_x000001260	5.92035	0.00000	0.00000
		Hand	genus *Acidovorax*_x000003346	5.82432	0.00000	0.00000
		Hand	*Acinetobacter* CF3_x000003901	5.51648	0.00000	0.00001
		Hand	genus *Pseudomonas*_x000001350	5.34174	0.00000	0.00002
		Hand	order *Rickettsiales*_x000000045	5.01316	0.00000	0.00011
		Hand	genus *Prevotella*_x000003689	4.81895	0.00000	0.00029
		Hand	*Streptococcus peroris*_x000004734	4.76201	0.00000	0.00039
		Hand	*Bacillus thuringiensis*_x000004033	4.74917	0.00000	0.00041
		Hand	*Pseudomonas* M9J918_x000005540	4.42942	0.00001	0.00187
		Hand	*Burkholderia* NTUIOB_TPF6_x000003353	4.37466	0.00001	0.00240
		Hand	*Veillonella* ADV_269.01_x000004149	4.12510	0.00004	0.00726
		Hand	genus *Corynebacterium*_x000004680	4.07743	0.00005	0.00888
		Hand	genus *Granulicatella*_x000004028	3.73856	0.00019	0.03517
		Hand	family *Neisseriaceae*_x000003348	3.73854	0.00019	0.03517

For porous versus non-porous analysis, a negative W-statistic indicates the taxa was enriched in non-porous samples, whereas a positive W-statistic indicates the taxa was enriched in porous samples (highlighted in gray). For hand versus foot-contacted surface analysis, a negative W-statistic indicates the taxa was enriched in foot-contacted, whereas a positive W-statistic indicates it was enriched in hand-contacted samples (light gray). Number starting with x after taxa names indicates a unique identifier assigned by GAST.

Complete bioinformatics analysis is included in the data supplement (add URL here).

## Discussion

To the best of our knowledge, this is the first study to broadly screen for both bacterial and fungal load and bacterial taxa and to specifically investigate the sources of contamination in an outpatient REHAB clinic. Findings refute the study hypothesis: total bacterial contamination was not correlated to patient contact. The study highlights include the finding that bacterial and fungal contamination on clinic surfaces was most closely correlated with the type of surface and how that surface was contacted rather than the degree of contact or frequency of cleaning. Findings suggest the combination of surface type (porous versus non-porous) and how the surface is contacted (hand versus foot-contact) likely plays a role in determining the make-up of the microbiome. Our findings suggest that non-porous surfaces such as metal and polished vinyl are more resistant to contamination in terms of overall number of microbes compared to porous surface, such as foam mats and handles of exercise equipment including weights, bikes and treadmills. An interesting finding was that non-porous and porous surfaces harbor different microbial communities. For example, non-porous surfaces were higher in the genus *Pseudomonas* whereas porous surfaces were higher in *Corynebacterium*. Moreover, surfaces primarily contacted by hand harbored a different microbiome compared to surfaces contacted primarily by foot. Foot-contacted surfaces were enriched in *Rickettsiales*, *Bacillus*, *Lachnospiraceae*, and *Bartonella* taxa, whereas hand-contacted surfaces were enriched in members of the *Corynebacterium*, *Streptococcus* and *Actinomyces* genera.

Foot contacted surfaces were contaminated with microbes related to zoonotic infections. For example, the genus *Bartonella* was one of the top 5 microbes enriched in foot-contacted surfaces and 13 species of this genus are associated with zoonotic infections [23]. On the other hand, *Streptococcus* mitis was among the top 5 most enriched microbes on hand-contacted surfaces and this bacteria is associated with the oral cavity, dental caries and oral and skin ulcerations [24]. The significance of this is that each surface may support potentially different pathological taxa depending on surface type and how contacted.

The above finding are related to the findings by Lax et al. [25]. The Lax et al. study examined the microbiome of a large university hospital from prior to opening, through one year [25]. This study did not directly examine how hospital surfaces were contaminated but did find that the surface microbiome of a patient’s room increasingly came to resemble the skin microbiota of the occupant with increased length of stay [25]. The occupant’s skin microbiota was the overwhelming factor in surface contamination, indicating that direct or indirect contact was involved in seeding these surfaces. An interesting finding from Lax et al. was that the occupant’s ambulatory status was the only other factor that significantly altered the room’s microbiome [25]. Although the Lax et al. study was conducted in hospital rooms and an outpatient REHAB clinic was the subject of the current study, a similarity is that the overall microbiome of the REHAB clinic (Fig 4B) also resembled the general human skin microbiota. Of the six most common genera of bacteria found on the REHAB clinic surfaces, comprising >78% of bacteria, 5 were common commensal microbes found on the skin (the exception being *Acinetobacter*) [25]. This finding suggests that staff or patients directly or indirectly seed these surfaces, but likely other factors such as the type of surface and efficacy of cleaning (which is likely different than frequency of cleaning) may also be involved.

A study by Gontjes et al. surveyed the microbiome of nursing home rehabilitation gyms and specifically examined transmission of microbes from surfaces to people and vice versa [6]. This study found multidrug resistant microbes on many pieces of gym equipment and concluded that these common spaces are repositories for antibiotic resistant microbes that are transmitted about 17% of the time from surfaces to patients or staff [6]. Unlike the Gontjes et al. study, we did not look at the sequence of contamination, from patients to surfaces or vice versa; therefore, our study design did not allow for direct analysis of transmission. The finding that porous surfaces harbor more and potentially different and problematic microbes such as *Staphylococcus aureus* compared to non-porous surfaces suggests that these surfaces may also be microbe reservoirs in an outpatient REHAB clinic.

Other studies also suggest that specific surfaces in a hospital room are key to the environmental microbiome and contribute to spread of infection [24–30]. A review by Weber et al. examined factors associated with common pathogens in the hospital room environment [26]. They found that common pathogens in hospitals can survive on surfaces for prolonged periods depending on the type of microbe and the local conditions [26]. For example, *Acinetobacter*, which was present at a relatively high level on some REHAB clinic surfaces in our study (comprising 5.97% of the total), can survive for up to weeks on moist surfaces [24]. This microbe can develop drug resistance and has been shown to be responsible for outbreaks in hospital and intensive care units with high mortality [24]. Two studies identified that both high and low touch surfaces contribute to the hospital room microbiome. Donskey et al. identified portable equipment and floors as highly contaminated surfaces [30] and Deshpande et al. identified floors as a key area in microbe transmission [28], even though they were not the most commonly contacted surfaces. This is consistent with our finding, indicating that the type of surface is related to the type of contamination.

Another finding from the current study is that there were potentially pathological microbes present on the REHAB clinic surfaces based on the presence of genus classification. The World Health Organization published a list of pathogenic microbes of heightening concern in 2017 because of their association with HAIs, potential for high mortality and propensity for acquiring antibiotic resistance [31]. This list includes members of *Acinetobacter*, *Pseudomonas* and *Enterobacteriaceae* [31]. In addition to *Staphylococcus aureus*, all three of these taxa were present on surfaces in the REHAB clinic sampled in this study, in addition to *Bartonella* on foot-contacted surfaces. While we do not know which species were represented, there are multiple potentially pathogenic members of these genera, and we can speculate that pathogens may also be present on surfaces in many other outpatient REHAB clinics due to their common occurrence. This highlights the need to determine how both the level and means of transmission of these microbes in outpatient REHAB clinics can be controlled.

One area that may require greater exploration is examining the nature of clinic surfaces. These results suggest that the type of surface is important in not only the amount, but also the type of bacteria harbored there. While this may seem obvious for some categories of surface such as metal and vinyl or countertops that are easily cleaned, it is less obvious for others, such as the rubber gym ball, and handles of the treadmill and bike. Surfaces that harbor bacteria are generally more difficult to clean thoroughly with cleaning products due to the porous nature of the material. As shown by two-way ADONIS analysis of QIIME data, the combination of frequency of contact and type of surface are related to the nature of the surface microbiome. A surface must be seeded before it can be colonized, but likely our measurement of “frequency of contact” did not capture the efficacy of microbe transmission since not all contacts are equal in terms of duration, intimacy and degree of contact of surfaces.

Cleaning or sanitization strategies may need to be developed to address the differences in surface porosity. This could include different cleaning agents, allowing for a longer period of cleaning agent contact, using disposable covers for certain clinic surfaces or changing the surface materials altogether. Another area for future research is going beyond frequency of cleaning and contact and examining the degree of surface contact and the efficacy of cleaning.

Outpatient facilities in general, and outpatient REHAB clinics specifically, have not been well studied as sources for HAIs. Exercise equipment and mats are constantly shared throughout the day at outpatient clinics, similar to the situation with sports like wrestling, offering a source of transmission for infections such as MRSA [32]. While not studied specifically in REHAB clinics, exam or treatment tables in other types of outpatient clinics have been found to be contaminated with MRSA and were listed as high traffic surfaces [33]. The types of tables examined in the Markey and Stevens study included examination tables in general medicine, oncology and hemodialysis clinics, but may be comparable in terms of the degree of patient contact to treatment tables and high-low mat table in an outpatient REHAB clinic [33]. Because many outpatient care settings are not certified by Centers for Medicare and Medicaid Services, or licensed by states, they may not use the same robust infection control programs as are used in inpatient facilities [33].

### Limitations

This was an observational pilot study, which contributed to several limitations with the project. The first limiting factor was the relatively short observation period in comparison to the total amount of contact at the clinic. Observation was only done for 20 hours in total, although the clinic had been open for 40 hours a week x 50 weeks per year for the last 3 years at the time of the study. Given 11 surfaces had no contact, there may have been a floor effect. A larger observation period may have augmented the power of the study to detect a contact effect.

The second limitation of this study is the limited population observed at the clinic, as the facility was a small outpatient REHAB clinic. Patients at this clinic were often more medically stable and presented with only musculoskeletal injuries. A third limitation was related to lack of culturing bacteria and fungi to supplement the sequencing data. Utilizing solely 16S rRNA sequencing may have missed under-represented or over-represented important microbes only detectable by culturing. Some significant pathologic microbes may have been present below the detectable threshold using 16S rRNA sequencing (too few reads for inclusion), but may have been detected by culturing. Alternately, some microbes may not have been viable, and yet their DNA was still present and was therefore included in qPCR and sequencing results. The significance of high and low levels in terms of risk of disease (i.e., if there was a high level of bacterial DNA, does that mean there was a high risk of contracting an infection?) is unclear at this point. Another limitation is that surfaces were only observed for frequency of contact and the degree or intimacy of contact was not measured. Also, we did not control for the area of the surface swiped (i.e., different samples represented different total areas).

### Conclusion

This observational pilot study aimed to describe the microbiome of an outpatient REHAB clinic and to determine which factors contributed to contamination. Frequency of contact was not related to contamination, but rather the type of surface or porosity of surfaces and the way they are contacted in a REHAB clinic may play an underestimated, but important, role in contamination and the types of microbes present. Based on these findings, it is important to consider the surfaces within the REHAB clinic in order to ensure the best sanitization or cleaning protocols are chosen to help reduce overall contamination.

## Supporting information

S1 Fig (JPG)

S1 Table (XLSX)
