# Supplementary figures and images for: The microbiome of an outpatient rehabilitation clinic and predictors of contamination: A pilot study
Source: PLoS One. 2023 May 4;18(5):e0281299. doi: 10.1371/journal.pone.0281299 (PMC10159339; doi:10.1371/journal.pone.0281299)

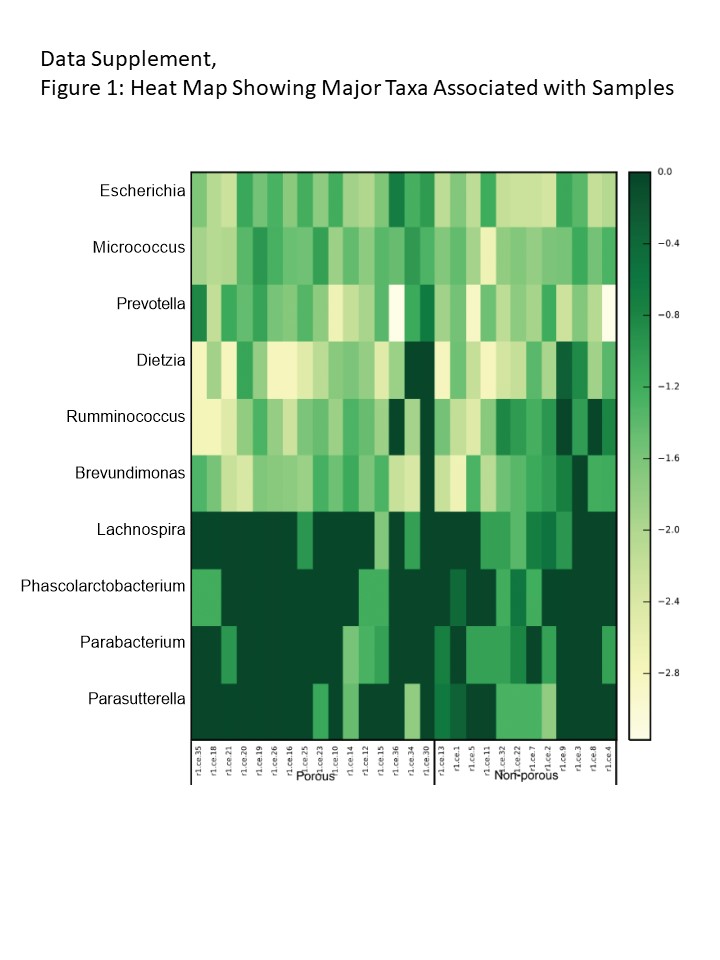

Supplement: S1 Fig — (JPG) [file pone.0281299.s001.jpg]
